# Oropharyngeal administration of mother’s colostrum, health outcomes of premature infants: study protocol for a randomized controlled trial

**DOI:** 10.1186/s13063-015-0969-6

**Published:** 2015-10-12

**Authors:** Nancy A. Rodriguez, Maximo Vento, Erika C. Claud, Chihsiung E. Wang, Michael S. Caplan

**Affiliations:** NorthShore University HealthSystem, Evanston, IL USA; University of Chicago Pritzker School of Medicine, Chicago, IL USA; Neonatal Research Group, Health Research Institute La Fe, Valencia, Spain; Clinician Researcher, Pritzker School of Medicine, Neonatal Nurse Practitioner, NorthShore University HealthSystem, 2650 Ridge Ave, Evanston, IL 60201 USA

**Keywords:** Breast milk, Human milk, Mother’s milk, Colostrum, Oropharyngeal, Oral immune, Oral care, Premature, Extremely low birth weight, Very low birth weight, Oral immune therapy

## Abstract

**Background:**

Extremely premature (birth weight < 1250 g) infants are at high risk for acquiring late-onset sepsis and necrotizing enterocolitis, which are associated with significant mortality and morbidity. Own mother's milk contains protective (immune and trophic) biofactors which provide antimicrobial, anti-inflammatory, antioxidant, and immunomodulatory functions, enhance intestinal microbiota, and promote intestinal maturation. Many of these biofactors are most highly concentrated in the milk expressed by mothers of extremely premature infants. However, since extremely premature infants do not receive oral milk feeds until 32 weeks post-conceptional age, they lack the potential benefit provided by milk (biofactor) exposure to oropharyngeal immunocompetent cells, and this deficiency could contribute to late-onset sepsis and necrotizing enterocolitis. Therefore, oropharyngeal administration of own mother's milk may improve the health outcomes of these infants.

**Objectives:**

To compare the effects of oropharyngeal administration of mother’s milk to a placebo, for important clinical outcomes, including (1A) reducing the incidence of late-onset sepsis (primary outcome) and (1B) necrotizing enterocolitis and death (secondary outcomes). To identify the biomechanisms responsible for the beneficial effects of oropharyngeal mother’s milk for extremely premature infants, including; (2A) enhancement of gastrointestinal (fecal) microbiota (2B) improvement in antioxidant defense maturation or reduction of pro-oxidant status, and (2C) maturation of immunostimulatory effects as measured by changes in urinary lactoferrin.

**Methods/Design:**

A 5-year, multi-center, double-blind, randomized controlled trial designed to evaluate the safety and efficacy of oropharyngeal mother’s milk to reduce the incidence of (1A) late-onset sepsis and (1B) necrotizing enterocolitis and death in a large cohort of extremely premature infants (*n* = 622; total patients enrolled). Enrolled infants are randomly assigned to one of 2 groups: Group A infants receive 0.2 mL of own mother's milk, via oropharyngeal administration, every 2 hours for 48 hours, then every 3 hours until 32 weeks corrected-gestational age. Group B infants receive a placebo (0.2 mL sterile water) following the same protocol. Milk, urine, oral mucosal swab, and stool samples are collected at various time points, before, during and after the treatment periods. Health outcome and safety data are collected throughout the infant’s stay.

**Trial registration:**

ClinicalTrials.gov identifier: NCT02116699 on 11 April 2014. Last updated: 26 May 2015

## Background

Despite advances in neonatal medicine, extremely premature (birth weight < 1250 g) infants have substantial mortality and morbidity, often resulting from infectious morbidities including late-onset sepsis (L-OS) and necrotizing enterocolitis (NEC) [1–8].

L-OS is highly prevalent (32–53 %) [6, 7], and costly (approximately US $23,317 per episode) [8] for this population. NEC accounts for an estimated US $6.5 million in additional hospital costs yearly, in the United States [3]. The risk for acquiring NEC is inversely related to birth weight; therefore, extremely premature infants have the highest incidence (10–12 %) of NEC, as well as the highest NEC-associated mortality rates [4]. With increased survival of premature infants, even a modest reduction in the incidence of L-OS and NEC could yield a substantial cost savings, and improved health outcomes for these infants. Minimizing these devastating infections takes on a greater urgency – *starting with the first days of life.*

L-OS is common in extremely premature infants because they are functionally immunodeficient, have an immature intestinal mucosal barrier [9–11], and require multiple invasive lines as part of their care. Pathogenic (intestinal) bacteria are able to translocate across the immature epithelial barrier and gain access to the bloodstream, while invasive catheters allow pathogenic bacteria a simple point of entry into the circulation. While Gram-positive organisms (particularly coagulase-negative *Staphylococcus* spp.) tend to be the predominant pathogens causing L-OS in this population, Gram-negative bacilli account for up to 30 % of episodes [12].

Compared to larger infants, extremely premature infants are more frequently exposed to invasive procedures and remain hospitalized in the pathogen-laden neonatal intensive care unit (NICU) for a prolonged duration, typically between 12–17 weeks. The long-term exposure to pathogenic organisms in over-crowded NICU conditions, overuse of antibiotics, delayed initiation of enteral feeds, and presence of nasogastric tubes (NGTs) and suction catheters are factors that decrease microbial diversity and promote abnormal microbiota [13], promoting pathogen translocation with subsequent L-OS. The immature gastrointestinal tract makes the tolerance to enteral feeds problematic, and this necessitates the long-term presence of an indwelling central venous catheter for the provision of intravenous parenteral nutrition, factors which significantly increase the risk of L-OS.

The pathogenesis for L-OS is, therefore, multifactorial, and studies suggest that lactoferrin supplementation [14], early removal of invasive catheters and exposure to human milk feedings can lower the risk [6, 7]. Lactoferrin is a glycoprotein with potent anti-microbial, anti-inflammatory, anti-oxidant and immunomodulatory functions [15–17]. It is contained in mother’s milk (especially colostrum), and highly concentrated in the milk expressed by women who deliver extremely premature infants [16–18]. A recent multi-center randomized controlled trial (RCT) [14] showed that preterm infants who received exogenous (bovine) oral lactoferrin supplementation had a significantly (50 %) reduced incidence of L-OS (9/153; 5.9 %) compared to the placebo-treated control group (29/168; 17.3 % (relative risk , 0.34; 95 % CI, 0.17–0.70; *P* = 0.002). Importantly, researchers utilized a dosage of lactoferrin similar to concentrations naturally found in human breast milk.

NEC, like L-OS, is associated with significant mortality and long-term morbidities for extremely premature infants. NEC is an inflammatory bowel necrosis which involves mucosal injury, altered intestinal microbiota, an unbalanced pro-inflammatory response, and abnormal host defense [4]. NEC is common in extremely premature infants because they have an immature intestinal epithelial barrier that is vulnerable to injury, a gastrointestinal microbiota which has a predominance of potential pathogens, an unbalanced pro-inflammatory response to pathogenic bacteria by immature enterocytes, decreased tight junctions between epithelial cells (permitting the translocation of pathogens) and an immature host defense system [4, 5, 13].

Pathogenic colonization of the preterm gut appears to be a primary step in the pathogenesis of both L-OS and NEC [5, 13, 19–21]. A pathogen-predominant microbiota promotes injury to the mucosal barrier and facilitates bacterial translocation from the gut into the bloodstream [22]. Recent research suggests that specific pathogens colonize the preterm gut before they invade the bloodstream with subsequent L-OS, confirming the importance of abnormal microbiota with subsequent mucosal translocation as an important pathomechanism [22, 23]. Therefore, optimizing the intestinal microbiome in extremely premature infants could reduce the risk of L-OS, and also NEC. Methods to enhance beneficial bacteria and improve microbial diversity may decrease the risk for L-OS and NEC via multiple mechanisms. Exposure to mother’s milk post-birth can reduce colonization by pathogenic organisms and increase commensal bacteria, reducing bacterial translocation, modulating the inflammatory response and decreasing intestinal injury [24–27].

Mother’s milk feedings have been linked with improved health outcomes for extremely preterm infants, including a lower incidence and severity of L-OS, NEC and enhanced neurodevelopmental outcomes [6, 28–33]. This protection is attributed to a multitude of protective (immune and trophic) biofactors, contained in mother’s milk. Many of these biofactors are more highly concentrated in the milk expressed by women who deliver extremely premature infants, particularly in colostrum (early milk), and are also contained in amniotic fluid. These biofactors include lactoferrin, immunoglobulins, growth factors, hormones, enzymes, antioxidants, nucleotides, polyunsaturated fatty acids, erythropoietin, lysozyme, anti-inflammatory cytokines and oligosaccharides [28–47]. Collectively, milk biofactors protect against L-OS and NEC, because of their ability to provide antimicrobial, anti-inflammatory and immunomodulatory functions, inhibit pathogen adhesion to the gastrointestinal mucosa, enhance gastrointestinal microbiota, maintain the integrity of the intestinal barrier and repair areas of injury, promote intestinal maturation and motility, and provide antioxidant protection [24–47]. The ability for mother’s milk to promote an optimal gastrointestinal microbiota, and stimulate healthy intestinal homeostasis, is fundamental to its ability to protect infants against L-OS and NEC [48].

Many biomechanisms likely contribute to mother’s milk benefits, but based on compelling evidence and our own preliminary data, we suggest that mother’s milk effects on microbiome, antioxidant defense, and immunostimulatory lactoferrin are inter-related and together contribute to a reduced incidence of infection and attenuated inflammatory responses. For example, recent studies suggest that pro-oxidant status can directly injury the intestinal epithelium and also disrupt the intestinal microbiome [49]; increasing the risk for L-OS and NEC. Lactoferrin, one of many biofactors contained in mother’s milk, can protect against L-OS and NEC via various mechanisms. First, lactoferrin has prebiotic and bifidogenic actions, promoting the growth of healthy commensals such *as Bifidobacteria* spp. and *Lactobacilli* spp. [50]. Second, lactoferrin has maturational effects on the intestine; promoting proliferation, growth and maturation of enterocytes, and closing enteric gap junctions [15]. Third, lactoferrin directly protects the intestinal epithelium from injury due to oxidative stress [51] and inflammation [52], and also prevents pathogen adhesion to the epithelial barrier, preventing (pathogen) translocation into the bloodstream. Finally, lactoferrin modulates cytokine production through direct contact with enterocytes and gut-associated lymphoid tissues [15, 52–55], down-regulating pro-inflammatory mediators which can injure the intestinal mucosa. Through these various mechanism, lactoferrin promotes a healthy microbiome, protects the intestine against injury, and prevents bacterial translocation into the bloodstream, preventing both L-OS and NEC. Understanding these important mechanisms may not only provide information to better understand the pathomechanisms for L-OS and NEC, but may also lead to important new approaches for prevention and treatment.

The milk expressed by women who deliver extremely premature infants is more highly concentrated in many protective biofactors – also present in amniotic fluid – compared to milk expressed at term [16, 17, 56–65]. Colostrum (early milk) is especially protective [37]. While the link between mother’s milk feedings and a decreased incidence and severity of infection is well-established, these gestation-specific trends in composition may offer additional protection against infection, for the extremely premature infant during the first weeks of life [66]. However, clinical instability typically precludes enteral feeds for extremely premature infants in the first days of life. Once started, enteral feeds are given via a NGT which bypasses the infant’s oropharynx. Therefore, the infant’s oropharynx is not exposed to protective (immune and trophic) milk biofactors until per oral feeds are introduced several weeks post-birth [48]. *If mother’s milk is not available and formula is given, then the infant’s oropharynx will never be exposed post-birth to protective biofactors* [48]. In a normal term pregnancy, the fetus’s oropharynx is continually exposed to protective immune and trophic biofactors, which are present in amniotic fluid, until 40 completed weeks of gestation. *We theorize that the lack of oropharyngeal exposure to protective biofactors post-birth for extremely premature infants, may be contributing substantially to many prematurity-associated morbidities including L-OS and NEC*.

The particular influence of early (mother’s) milk exposure to the extremely premature infant’s oropharynx is unique and important. Experts postulate that breastfed infants are protected against infection because of the immunostimulatory effects of protective breast milk biofactors (including cytokines) on the infant’s oropharyngeal-associated lymphoid tissue (OFALT) [67, 68]. Thus, the exposure of milk biofactors to oropharyngeal immunocompetent cells appears to be protective and highly beneficial for the infant. While the term breastfed infant benefits from this protection, the extremely premature infant does not, and oral feeds are not introduced until several weeks post-birth [48]. Oropharyngeal administration of mother’s milk [48, 66, 69, 70], may serve as a natural alternative to provide a continuum of protective effects post-utero for extremely premature infants [48].

With oropharyngeal administration, milk drops are placed directly onto the infant’s oral mucosa [66, 69], so that biofactors may provide immunostimulatory effects [66–70]. Using this approach, we theorize that oropharyngeal administration of own mother's milk is protective against L-OS and NEC [48] via several mechanisms: 1) interaction of milk cytokines with oropharyngeal immune cells, 2) mucosal absorption of protective biofactors, 3) barrier protection against pathogens, 4) local and systemic effects of oligosaccharides which modulate intestinal microbiota and 5) beneficial effect of protective antioxidants. Milk cytokine interaction with OFALT may provide immune stimulation. Mucosal absorption of (milk) lactoferrin may result in higher concentrations of urinary lactoferrin suggestive of systemic immune protection against L-OS. A recent small (*n* = 48) RCT [71], which was designed to determine the immunologic effect of oropharyngeal colostrum administration, showed higher concentrations of urinary lactoferrin for milk-treated extremely premature infants at 1 week of age, compared to placebo-treated controls (3.5 versus 0.9 μg/g creatinine *P* = 0.01). This finding is consistent with our own pilot data [70], and suggests that lactoferrin may be absorbed via the mucosa with passage into the circulation, and then excreted into urine following oropharyngeal milk therapy [71]. During oropharyngeal administration of mother’s milk, intestinal growth factors may also be absorbed mucosally or may travel to the gut and accelerate intestinal maturation. Oligosaccharides may be absorbed mucosally with systemic effects, or may travel to the gut where they can exert prebiotic effects enhancing *Bifidobacteria* spp. growth, and also serve as decoy receptors to competitively bind and inhibit pathogens, preventing translocation and L-OS. Barrier protection (provided by lactoferrin and secretory immunoglobulin A) prevents pathogen adherence to epithelial cell surfaces in the gastrointestinal tract, protecting against both L-OS and NEC. Antioxidants protect immune cells from injury, prevent oxidative stress-induced changes in microbiota which promote pathogenic species and thereby reduce inflammation and injury to the intestinal epithelium, preventing translocation and protecting against L-OS and NEC. Milk macrophages may also travel to the gut, where they can survive for up to a week and secrete intestinal growth factors and anti-inflammatory cytokines [63].

Our previous studies [69, 70] established feasibility for this technique and results were suggestive of immunostimulatory effects [70]. Recent research from other investigators suggest additional benefits including protection against ventilator-associated pneumonia, L-OS, NEC and clinical sepsis, an earlier attainment of full enteral feeds, enhanced maturation of oral feeding skills, improved growth, enhanced immune function, and improved breastfeeding outcomes [71–81]. An earlier attainment of full enteral feeds has been reported in several studies [70, 72, 76], and has important implications in terms of L-OS risk. When full enteral feeds are reached sooner, there is earlier removal of centrally placed venous catheters and intravenous parenteral nutrition, significantly reducing the infant’s risk for acquiring L-OS. Findings from these published reports suggest that oropharyngeal administration of mother’s milk may be beneficial for extremely premature infants. However, significant limitations, including small samples, retrospective analysis, and inconsistency in the technique, limit generalizability.

Published reports are primarily from small retrospective cohort studies, feasibility trials, studies in which oropharyngeal colostrum was included as part of a standardized feeding protocol, and a recent small (*n* = 48) RCT [71]. In published reports, investigators used various terms to describe ‘oropharyngeal administration of milk’ including oral care, mouth care, oral swabbing, oral colostrum and oral immune therapy. However, the underlying premise was the same, placing drops of mother’s milk (including early milk: colostrum) into the infant’s mouth so that biofactors may stimulate the infant’s oropharyngeal-associated lymphoid tissues, providing protective immunomodulatory effects [48].

In published reports, there has been wide variation in the technique, including significant variation in the dose of milk administered (ranging from 0.1 mL to 1.0 mL), in the frequency of treatments (every 2 to 6 hours and also on an “as needed” basis), and in the duration of the treatment protocol (from 2 to 7 days). Some investigators used syringes to draw up a precise volume of mother’s milk, while others utilized a cotton swab soaked with mother’s milk. Repeatedly dipping a swab into a container of mother’s milk can contaminate the milk with NICU pathogens and increase infection risk for the infant. The use of a cotton swab increases the risk that (cotton) fibers may be released during the administration procedure and potentially aspirated. Importantly, a cotton swab has been shown to absorb up to 97 % of the milk during 10 seconds of swabbing. Administering a precise volume with a sterile syringe, as opposed to soaking a swab in the milk, minimizes the absorption of the milk into the swab. In this manner, more milk remains on the mucosa for a consistent “dose.”

We have tested many approaches but for this study, we chose to deliver a precise (small volume) “dose” (using a sterile syringe) followed by gently and brief buccal swabbing with a small swab which has a polyurethane foam head. The buccal swabbing serves to evenly distribute the milk over the mucosa, but is done quickly (≤5 seconds each side) and gently (with a non-cotton swab) in order to avoid unnecessary friction and irritation to the fragile mucosal tissues.

In published reports, infants received colostrum, but not mature milk, and the duration of treatment was brief, ranging from 2 to 7 days. In our pilot studies, infants also received only colostrum (not mature milk) for the intervention, and the treatment period was only 48 hours in duration. However, for this current trial, infants randomized to the treatment group receive colostrum and also mature milk, because we recognize that (preterm) mature milk remains highly concentrated in many protective biofactors. Since our premise is that oropharyngeal administration of mother’s milk may serve to mimic the protective effects of amniotic fluid biofactors in the premature infant’s oropharynx [48], enrolled subjects receive frequent “doses” of mother’s milk (or placebo) until 32 weeks corrected gestational age (CGA); when per oral feeds can be safely introduced.

In summary, the oropharyngeal administration of mother’s milk appears to be beneficial for extremely premature infants; however, safety and efficacy have not been established. While emerging evidence is promising, small samples and wide variation in the technique limit generalizability. An adequately-powered RCT is, therefore, needed to set the standard for this technique, and to definitively establish the safety and efficacy of this intervention for extremely premature infants.

## Methods/Design

### Study design

**Fig. 1:**
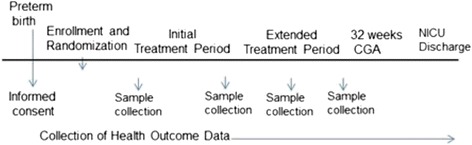
Timeline of the study. After informed consent is obtained, the infant is enrolled, randomized, and begins receiving treatments. The treatments are given until the infant reaches 32 weeks CGA. Health outcome data is collected throughout the infant’s hospitalization until NICU discharge. CGA, corrected gestational age; NICU, neonatal intensive care unit

This study is a 5-year, prospective, double-blind, placebo-controlled randomized clinical trial across 5 large neonatal centers, designed to evaluate the safety and efficacy of oropharyngeal administration of own mother's milk to reduce the incidence of L-OS, and NEC and death, in a large cohort of extremely premature infants (*n* = 622; total patients enrolled). A multi-center 5-year trial is necessary in order to reach the target sample, based on average numbers of extremely premature infants admitted yearly to participating sites. Enrolled infants are randomized to one of 2 groups using computer-generated random numbers: Group A (treatment) infants receive 0.2 mL of own mother’s milk (OMM) via oropharyngeal administration every 2 hours for an initial treatment period of 48 continuous hours, followed by an extended treatment period of 0.2 mL of OMM via oropharyngeal administration every 3 hours until 32 weeks CGA. Group B infants (control) receive a placebo (sterile water, and blinded as described below) using the same dose and following the same protocol. Samples of mother’s milk and infant’s urine are collected at baseline and within 6 hours after the completion of the Initial Treatment Period and the Extended Treatment Period. A sample of infant’s urine is collected at 7 days of life. Milk, oral mucosal swab, and stool samples are collected at the time of first stool, 2 weeks of life and at 32 weeks CGA. For infants diagnosed with L-OS or NEC, a stool sample is collected at the time of diagnosis. Health outcome and safety data are closely monitored and collected throughout the infant’s stay until discharge or death. See Fig. 1 for the timeline of the study.

#### Study approval

The study was approved by the Institutional Review Board (IRB) at the NorthShore University HealthSystem (primary site) and by the IRBs at the New Hanover Regional Medical Center and the St. Joseph’s Regional Medical Center. Subject enrollment is in progress at these three study sites. Recruitment at the Advocate Children’s Hospital and also the Morristown Medical Center study site will not begin until IRB approval has been obtained. All investigators, treating physicians and nurses, project director, data analyst, and lab technicians are blind to group assignment, except the (site) research nurse who assigns subjects into groups and prepares syringes.

#### Study site and population

Infants are being recruited from 5 NICUs in hospitals within the United States: (1) NorthShore University HealthSystem in Evanston, IL, (2) Betty H. Cameron Women & Children's Hospital (New Hanover Regional Medical Center) in Wilmington, NC, (3) St. Joseph’s Children’s Hospital in Paterson, NJ; (4) Advocate Children’s Hospital in Park Ridge, IL (recruitment will not begin at this site until IRB approval is obtained) and (5) Morristown Medical Center in Morristown, NJ (recruitment will not begin at this site until IRB approval is obtained). We anticipate a study population comprised of the following demographics: 31 % African American, 35 % Non-hispanic Caucasian, and 27 % Hispanic, 3 % Asian and 4 % Other.

#### Inclusion/exclusion criteria

Extremely premature infants are eligible for the study if they meet the following inclusion criteria: birth weight < 1250 g; mother plans to pump and provide milk for at least 2 months; absence of severe congenital anomalies; NICU admission ≤ 24 hours of life; ability to begin protocol within 96 hours of life. The exclusion criteria are as follows: birth asphyxia (cord pH/arterial pH < 7.0); presence of a tracheoesophageal fistula, maternal + HIV status, maternal drug or substance use that precludes infant from receiving mother’s milk, triplets or higher order multiple births.

#### Participant recruitment, enrollment and informed consent

The collaborating sites have a relatively high census of extremely premature infants yearly and very high lactation initiation rates (80–90 %) which facilitates subject recruitment. Mothers on bed rest protocols in the high risk antenatal unit, who meet inclusion criteria (estimated fetal weight < 1250 g per prenatal ultrasound) are approached and invited to participate. In our pilot studies, this recruitment strategy was highly successful and the majority of women approached agreed to participate. Mothers who deliver without significant time in the antenatal unit are approached within 96 hours after delivery.

The principal investigator (PI) and/or research nurse explain the study in detail, answer questions, and leave the consent form so that the mother can read it and discuss the study with her family prior to enrolling. A second meeting is scheduled later that same day, when convenient for the mother, and the PI and/or research nurse return and answer any additional questions. Informed consent is obtained at this time (second meeting) if the mother decides to participate. This precaution is taken because these women are considered psychologically vulnerable, in terms of research. Mothers are informed that their care (or their baby’s care) will not be affected if they decide not to participate in this study. In addition, mothers are informed that they have a right to withdraw their infant (or themselves) from the study at any time and for any reason without compromise to their care, or their infant’s care.

If the woman is approached antenatally, before securing informed consent, the PI and/or research nurse explain to her that if she remains pregnant and the estimated fetal weight (EFW) is over 1250 g per prenatal ultrasound, then the infant, once born, will no longer qualify for the study. This is deemed a screen failure since the infant will no longer meet inclusion criteria. All women are assured that their infant’s care, and their breastfeeding support post-birth, will not be affected by their decision to participate in the study, or if they become ineligible. Since 27 % of enrolled mothers are expected to be Hispanic, Spanish-IRB-approved translations of study documents are available at all sites.

#### Randomization

A 1:1 blocked randomization scheme was utilized to yield “comparable” subjects in each of the 2 groups, stratified by birthweight (<750 g, 751–999 g, 1000–1250 g) within each site, to avoid imbalance in the distribution of the 2 groups at each site. We produced a separate block randomization list within each site for each subgroup (a total of three strata). The randomization is based on permutated blocks with random sizes varying from 2 to 4 (to avoid guessing) to make sure that each site will have an equal number of participants in each group. The randomization list was generated prior to study initiation using a random seed number via SAS PROC PLAN (SAS Inc., Cary, NC, USA) to generate the stratified randomization design. The statistician prepared sealed, numbered opaque envelopes for each site, with randomization assignment inside. The envelopes were given to the site’s research coordinator who assures that they are used in numerical order. After informed consent is obtained, the research coordinator opens an envelope, removes the group assignment form, writes the infant’s study ID # and date on the form, makes a copy of the form and sends it to the statistician, keeping the original in the infant’s study file. Group A (treatment) infants receive OMM, while Group B (control) infants receive a placebo (sterile water). In the case of a multiple birth, twins are stratified and randomized into either the intervention or the control group, and will be analyzed independently between the two experimental groups. Triplets or higher order multiple births are not be eligible for the study.

##### Retention

The major issue with retention is the need for mothers to provide milk until their infant reaches 32 weeks CGA. While most will be motivated to do so (based on our experience) we have conservatively estimated a 20 % dropout rate to account for mothers who discontinue pumping (based on previous experience). Only a very small volume (< ½ teaspoon) of milk is needed daily for the intervention and, as our pilot data shows that all mothers can readily express this amount, this should not be problematic.

#### Study procedures

##### Collection of milk

Research personnel within each site have been trained, using a standardized protocol (Protocol version 4; Dated 1/5/2015), to assure coherence of data collection. Mother’s milk samples are collected during the routine expression of milk, using a hospital-grade electric breast pump, and are stored in the NICU breastmilk refrigerator. When at least 2.5 mL (½ teaspoon) of mother’s milk is available, the research nurse prepares the syringes for the first 24 hours of the Initial Treatment Period. For infants in the placebo group, the milk is immediately frozen, in a separate NICU breastmilk freezer to maintain blinding, for later use when enteral feedings are started. Using sterile gloves, 24 sterile oral syringes are each filled with 0.1 mL of OMM or sterile water (based on group assignment), capped, and covered with a white tape as a blinding procedure. Each syringe is labeled with the patient’s name, medical record number, and the date and time of preparation. Syringes are prepared in the same sterile manner, by the research nurse, every 24 hours. For the Extended Treatment Period, a total of 16 syringes are prepared daily, for treatments to be given every 3 hours.

It is standard practice to encourage mothers of extremely premature infants to pump frequently (typically every 3 hours) in order to establish and maintain an abundant milk supply. Therefore, it is common for NICU nurses to see several containers, with varying amounts of mother’s milk, in the NICU refrigerator, on a daily basis. The milk is either used for enteral feeds or frozen for storage. Since only miniscule volumes of milk are needed daily for this intervention (2.4 mL and 1.6 mL per day, for Initial and Extended Treatment Periods, respectively) it has been our experience that NICU nurses do not notice that a small volume of milk has been removed from the refrigerator, and thus they remain blind to group assignment.

##### Oropharyngeal administration procedure

**Fig. 2:**
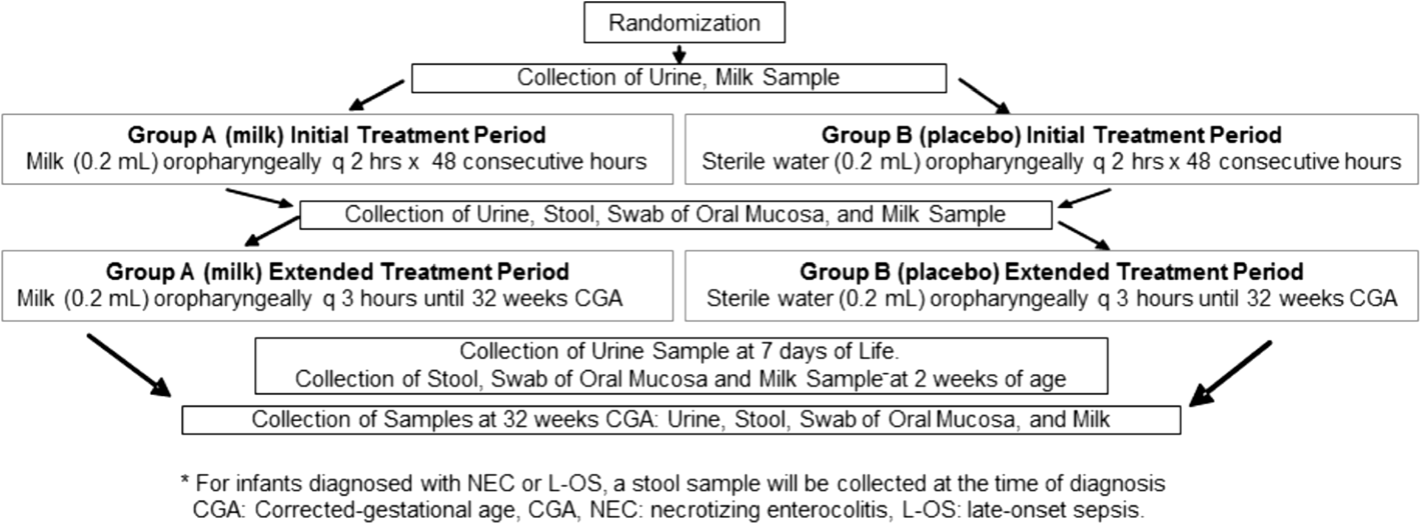
Study flow chart. Enrolled infants are randomized to receive either own mother’s milk or a placebo during the initial treatment period and the extended treatment period. Biological specimens (milk, urine, stool and swab of oral mucosa) are collected at specific time-points as depicted. CGA, corrected gestational age; L-OS, late-onset sepsis; NEC, necrotizing enterocolitis

Using a standardized protocol, the nurse provides the dosing as follows: two syringes are warmed to room temperature. The first syringe’s cap is removed and the tip of the syringe is gently placed into the infant’s mouth, alongside the right buccal mucosal tissue. The syringe tip is directed posteriorly towards the oropharynx, and the total volume (0.1 mL) is slowly administered, over at least 1 minute. The second syringe is placed in the infant’s mouth in the same manner, but alongside the left buccal mucosal tissue. The entire volume (0.1 mL) is administered slowly, over at least 1 minute. A petite swab is used to carefully swab the right buccal mucosal tissue, followed by the left buccal mucosal tissue (≤5 seconds each side). A total volume of 0.2 mL is administered per treatment, with buccal swabbing taking place over 10 seconds. Vital signs are carefully monitored throughout the procedure. The procedure is repeated every 2 hours for 48 consecutive hours during the Initial Treatment Period, which begins within the infant’s first 96 hours of life. The Extended Treatment Period begins immediately after the Initial Treatment Period has been completed and ends when the infant reaches 32 weeks CGA as depicted in Fig. 2. Dosing is provided every 3 hours during the Extended Treatment Period. Participation in this study does not interfere with the initiation or progression of enteral feedings, which is left to the discretion of the attending physician. Urine, oral mucosal swabs, and stool specimens are collected from enrolled infants, and milk samples from their mothers, at specific time points, as depicted in Fig. 2.

##### Objectives

Our specific Aim 1 is to compare the effects of oropharyngeal administration of mother’s milk to a placebo, for important clinical outcomes, including (1A) reducing the incidence of L-OS (primary outcome) and (1B) NEC and death. Our specific Aim 2 is to identify the biomechanisms responsible for the beneficial effects of oropharyngeal administration of mother’s own milk for extremely premature infants including (2A) enhancement of gastrointestinal (fecal) microbiota (2B) improvement in antioxidant defense maturation or reduction of pro-oxidant status, and (2C) maturation of immunostimulatory effects as measured by changes in urinary lactoferrin.

##### Sample size/Power analysis

We hypothesize that extremely premature infants who receive oropharyngeal administration of mother’s milk will have a significantly lower incidence of L-OS (primary endpoint). Based on recent (2012) data from the Vermont-Oxford Network that included > 20,000 ELBW infants, the incidence of L-OS in this population was 19.4 %. Based on data from Manzoni et al. [14], that showed a reduction of L-OS from 17 % to 6 % with exogenous bovine lactoferrin in preterm infants, we conservatively estimate a reduction in L-OS from 20 % to a total of 10 % in milk-treated infants. Since Manzoni et al. [14] used a lactoferrin dose similar to that in mother’s milk, and based on studies that mother’s milk reduces L-OS [6–8] this effect size of reduction (from 20 % to 10 %) should be realistic and obtainable given the patient population. Since many variables may alter the risk for L-OS in extremely premature infants multivariable logistic regression (for dichotomous L-OS outcome) will be performed adjusting for covariates such as sex, gestational age and birth weight. Power calculations were determined using PASS 14.0 software (NCSS, LLC, Kaysville, UT, USA). We estimate that up to a total of 10 covariates will account for a combined 20 % (*R*^*2*^ = 0.2) of variance in the regression model. Based on a 2-tailed alpha of 0.05 and 80 % power, a sample size of 498 (249 in each group) will be needed to reach a statistical power of 80 % to detect a difference of 10 % versus 20 % (effect size odds ratio (OR) = 0.44) in L-OS outcome between milk and placebo groups. Therefore, the study is adequately powered for the primary endpoint L-OS. The study sample is being accrued from 5 sites and, based on a lactation initiation rate of > 70 % for all sites (conservative, based on actual rates of 80–90 %), and an estimated refusal rate of 30 % (a conservative estimate based on our previous experience in recruiting subjects for this intervention), we will screen a total of 1270 patients to enroll 622, with an estimated dropout rate of 20 %, that will allow us a complete analysis for 498 subjects.

##### Outcome measures for specific Aim 1

Primary endpoints: L-OS is defined as the new onset of at least 2 clinical symptoms with a positive blood culture (noted after Day of life 3) and identification of an organism known to be a cause of sepsis rather than a contaminant. If the blood culture shows a potential contaminant (such as *Staphylococcus epidermidis*) a second blood culture is obtained to confirm the presence of bacteremia.

Secondary endpoints: NEC and death. NEC is defined according to modified Bell’s criteria stage > 2 with clinical signs and radiological evidence including any of the following: pneumatosis intestinalis, portal venous gas with or without pneumoperitoneum.

Covariates: sex, gestational age and birth weight will be included as covariates.

Safety endpoints: health outcome and safety data are closely monitored and collected throughout the infant’s stay until discharge or death. Infant safety is being assessed through the monitoring for adverse events by the Data Safety and Monitoring Board (DSMB), comprised of independent reviewers. The DSMB meets every 6 months during the first year of active enrollment, and thereafter at ¼, ½ and ¾ enrollment to ensure rapid identification of adverse events. Adverse events are defined to include aspiration, significant bradycardia following (oropharyngeal milk) administration leading to the need for resuscitation, and post-natal acquisition of cytomegalovirus (CMV) thought to be associated with fresh milk. Studies have identified the potential risk of milk-acquired CMV infection, but based on the small volumes and controversial risk (even with frozen milk), we promote fresh milk as our standard in this study, as we did with our pilot studies, and follow clinically for symptoms of acquired CMV and will intervene quickly if a case should arise. To maintain safety of enrolled infants, vital signs are carefully monitored during the oropharyngeal administration of the study substance (milk or sterile water). The nurses are instructed to immediately stop the procedure and to notify the site PI if any of the following signs of aspiration should occur: an increase in fraction of inspiratory oxygen > 0.1 to maintain a SpO_2_ > 85 %, bradycardia (heart rate (HR) < 100/minute), tachycardia (HR > 200/minute), tachypnea (respiratory rate (RR) > 80/minute), or apnea. Treatments are withheld during surgical and invasive procedures or based on clinical evidence of acidosis, NEC, multisystem organ failure, or other instances of clinical deterioration, but this decision is left to the discretion of the attending physician after discussion with the site PI (another neonatologist).

Built into this study, are interim analyses by the DSMB that will evaluate any significant adverse events that may require altering study protocol over the course of the study, and to analyze whether results with one arm are not significantly beneficial compared to the other. Adverse events could occur in the intervention and placebo groups (for bradycardia and aspiration). Ad-hoc DSMB meetings can be requested at any time if the PI or site investigators identify a potential safety issue. The PI is responsible for accurate documentation, investigation and follow-up of all study-related unanticipated serious events in a timely manner. To ensure that study-related unanticipated serious events are identified and managed expeditiously, the following reporting mechanism will be implemented: the PI (Dr. Rodriguez) will be notified immediately by the site investigator (collaborating neonatologist) of any study-related unanticipated and adverse events or serious safety issues. The PI will immediately request an ad-hoc DSMB meeting to review safety data. The DSMB will review the study-related unanticipated and adverse events and will make a determination as to whether the study should be continued, modified or terminated. The DSMB Chair will forward the report to the PI, who will submit the report to the NorthShore IRB and to site investigators (collaborating neonatologists) at each study site, who must, in turn, submit the report to their local IRBs. The report will include the DSMB’s recommendations to the IRB as to whether the study should be continued, modified or terminated. Any action taken by the IRB will be reported by the PI to the DSMB, and to site investigators and their IRBs.

##### Statistical analysis

Statistical analysis will be performed using an intention-to-treat (ITT) approach. Baseline infant and mother demographics and clinical characteristics will be summarized between milk and placebo groups using a chi-square or Fisher exact test for categorical variables (i.e. infant gender, race/ethnicity, socioeconomic status, maternal diagnosis of pre-eclampsia, premature and/or prolonged rupture of membranes, delivery mode, and steroid administration) and an independent *t* test or a Wilcoxon two-sample test for continuous variables (i.e. the time of initial exposure to mother’s own milk, duration [number of days] of central line placement and mechanical ventilation, gestational age, birth weight, post-natal steroids, antibiotic duration), depending on satisfying the normality assumption or not, respectively. A multivariable logistic regression with L-OS or NEC or death as the dependent variable, and group (milk versus placebo) as the primary independent variable will be constructed to assess the strength of association between oropharyngeal milk treatment and risk of L-OS or NEC or death, adjusting for pre-specified covariates including sex, gestational age and birth weight. Odds ratios and corresponding 95 % confidence intervals will be reported. Because the amount of mother’s milk that enrolled infants receive as a “feeding” will affect our study endpoints, we will closely monitor and record the total amount of mother’s milk received (percent of total feeding volume) for each study subject. We do not anticipate the amount of milk the infant receives as an enteral feed will be affected by group assignment. However, if we do observe a significant difference between the intervention and placebo groups, we will control for this variable in the regression models. While the main hypotheses will be tested using ITT analysis, we will also conduct per protocol analysis only for patients who complete the treatment. Completed treatment is defined as (the infant) having received 70 % of planned doses. However, this will be considered supplemental and exploratory, and the results will not be used as main conclusions of the study.

##### Analysis of safety endpoints

Safety endpoints will be analyzed using summary statistics (frequency, count, percent). We will compare each safety endpoint between milk and placebo groups using chi-square and Fisher exact tests. We will also analyze the safety endpoint count data by conducting Poisson regressions using group (milk versus placebo) as the independent variable, controlling for covariates described above if applicable.

##### Outcome measures for specific Aim 2

This is an exploratory aim of the trial and, therefore, should not be considered as the primary endpoint of the study. We hypothesize that oropharyngeal administration of OMM will modulate microbiome, antioxidant status and urinary lactoferrin concentrations for recipient infants. These measures are described below.

##### Microbiome

We hypothesize that mother’s milk will modulate fecal microbiota of extremely premature infants leading to increased microbial diversity with a *Lactobacillus/Bifidobacterium* predominance and decreased *Proteobacteria*. Samples of milk, oral mucosa swabs, and stool are collected at the time of first stool, 2 weeks of age and at 32 weeks CGA for enrolled subjects and frozen immediately at −80 °C. For subjects who develop L-OS or NEC an additional stool sample is obtained at the time of diagnosis, and results correlated with the 32 -week samples. The composition and structure of the microbiome from milk, oral mucosa, and stool will be tracked using high-throughput 16S rRNA-based gene analysis as per previously published protocols [20, 82].

##### Antioxidant status

We hypothesize that the oxidative stress associated with prematurity will be diminished with oropharyngeal administration of OMM, as measured by changes in levels of validated urinary biomarkers of oxidative stress, and this will correlate with the development of L-OS (late onset sepsis) and NEC (necrotizing enterocolitis).

Extremely premature infants are predisposed to oxidative stress-derived diseases because of (1) the need for oxygen supplementation in the first weeks of life with an overproduction of highly reactive oxygen-derived free radicals (FR) capable of causing damage to DNA, proteins and lipids; (2) immaturity of the antioxidant enzymatic and non-enzymatic defense system [83–89]. The deleterious action of free radicals upon proteins, lipids, and DNA can be assessed by determining highly specific biomarkers using ultra high performance liquid chromatography coupled to tandem mass spectrometry (UPLC-MS/MS) methods that have been validated using Food and Drug Administration (FDA) stringent requirements.

Our hypothesis is based on previous observations by our research team (Dr. M. Vento) showing that human milk-fed preterm infants have better antioxidant capacity compared to formula-fed cohorts [87]. Dr. Vento has established normative ranges for the measurement by UPLC-MS/MS of urinary biomarkers in extremely premature infants reflecting oxidative damage to proteins [87, 88]. His research has validated the whole spectrum of metabolites derived from phenylalanine (Phe) oxidation by non-physiologic pathways (orto, meta, nitro, 3Chlor-tyrosines) and oxidative damage to guanidine bases of DNA (8-oxo-dihydroxi-guanosine), and non-cyclo-oxygenase (COX) derived oxidative byproducts of arachidonic and docosahexanoic acid, essential components of cell membranes, in extremely premature infants such as F2-isoprostanes, specific Prostaglandins (PGs), Isofurans (IsoFs), Neuroprostanes (NeuPs) and Neurofurans (NeuFs).

The approach used to test this hypothesis will be the PGs and isoprostanes (IsoPs) in urine samples of the first 50 (25 milk, 25 placebo) enrolled infants, collected at 4 time points as depicted in Fig. 2. UPLC-MS/MS analysis is carried out on an Acquity-Xevo TQ system (Waters, Milford, MA, USA) using negative electrospray ionization. MS/MS detection is carried out by multiple reaction monitoring (MRM) employing specific acquisition parameters. In milk-treated infants, we anticipate lower elimination of oxidized tyrosines, oxidized bases of DNA and IsoPs and PGs compared to baseline. We expect urinary metabolites of the placebo-treated infants to be within the established ranges for the different metabolites.

##### Lactoferrin

We hypothesize that oropharyngeal administration of OMM will be associated with higher concentrations of urinary lactoferrin in treated infants. Our hypothesis is based on our pilot data [70], which showed higher concentrations of urinary lactoferrin in infants who received oropharyngeal administration of mother’s colostrum, compared to placebo-treated controls. No significant between-group differences were found; however, the sample size was small (*n* = 16). A large effect size (1.30) was noted for urinary lactoferrin, which suggests that results may have reached statistical significance if a larger sample had been used. This finding is consistent with research that has shown higher concentrations of urinary lactoferrin in mother’s milk-fed infants compared to formula-fed cohorts [90, 91]. Further studies have shown that the urinary lactoferrin is of maternal origin [92–94], although the precise mechanism of entry into the urine is unknown. The approach used to test this hypothesis will be the measurement of lactoferrin in urine samples at 3 time points (see Fig. 2), using a commercial ELISA assay. Lactoferrin concentrations will also be measured in milk samples from mothers, at the same three time points, to clarify whether the infant’s levels are due to the milk or from neonatal immune responses resulting from other factors. A mixed-effects regression model will be applied to assess whether the average at each time point or change over time is different between the two groups. The analysis will be done first for infant and mother separately, then an index variable will be created to define mother versus infant, and the difference between mothers and infants will be assessed. Appropriate variance-covariance structure will be determined by Akaike Information Criterion (AIC). Normality assumption will be evaluated using Shapiro-Wilk’s test or visualization of data distribution. Appropriate data transformation (i.e. log, square root) will be applied to correct the skewness.

##### Data management and quality control

The goals of data management are: (a) to ensure that data collected across sites are properly and accurately entered and documented; (b) to ensure that data will be stored in an electronic format that will allow the primary investigators of the project to retrieve data easily and to export data to statistical packages; and (c) to ensure the confidentiality of subjects. Prior to data collection and entry, a standard codebook was created which contains the variable names, descriptions, and value codes of each variable/item collected within each site. Following development of the codebook, a web-based SQL (Structural Query Language) data capture and archiving system was created using REDCap to standardize data collection across the five study sites. The web-based REDCap application, developed by Vanderbilt University, offers intuitive design and tailored clinical research workflows that enable research team members across collaborating study sites to share, capture and manage data. REDCap provides secure user-friendly web-based case report forms, real-time data entry validation (e.g. for data types and range checks) and audit trails. Users from different sites can be assigned different levels of access which can be form or rights based. All data are stored in the password-protected network server at NorthShore with back-up scheduled at every midnight. The original raw data within each site is stored in a locked cabinet accessed only by key project personnel. For quality control, we have developed and implemented standard protocols for data collection and enrollment monitoring across study sites. We have also implemented communication systems and documentation of study-related plans, decisions, progress, and analysis management for efficiently throughout all phases of the research. All research personnel within each site have been trained using a standard protocol. The training covers areas such as recruiting, enrollment, randomization, and web-based data collection.

The PI, Project Director and Data Manager conduct interim sites visits to collaborating study sites, to audit data for quality assurance, ensure protocol compliance and to address any issues. All IRB and Health Insurance Portability and Accountability (HIPAA) regulations are strictly enforced. The entire research team (including site PIs and research nurses) meet via conference call monthly, and as needed, to discuss enrollment, adherence to protocol, and to promptly address any issues.

##### Missing data

For missing data or gross outliers, we will use several strategies to handle missing, out-of-range, and potentially erroneous data (depending on the level of measurement of the variables) including considering patterns driven by demographics and behavioral factors to guide replacement decisions and replacing missing values with those generated through multiple imputation using SAS PROC MI (SAS Inc., Cary, NC, USA) procedure if missing data are MCAR (data are missing completely at random) or MAR (data are missing at random). If missing data are NMAR (not missing at random), we will use the “pattern mixture” approach to compute a “weighted average” of the parameters that are associated with the missing data to estimate what the data would have been.

## Discussion

This multi-center, double-blind RCT was designed to evaluate the safety and efficacy of oropharyngeal administration of OMM to reduce the incidence of L-OS and NEC in a large cohort of extremely premature infants. For treated infants, we anticipate beneficial gastrointestinal effects (e.g. trophic effects on the intestine and a predominance of beneficial microflora), immunostimulatory effects (higher concentrations of urinary lactoferrin), enhanced antioxidant status (measured by changes in levels of urinary biomarkers of oxidative stress), and a decreased incidence of L-OS and NEC.

To date, 77 subjects have been enrolled. There have been 11 screen failures because consent was obtained antenatally (per protocol), but once the mother gave birth the infant’s weight was over 1250 g and he/she was no longer eligible for the study. The remaining 66 subjects have completed treatment. A total of 13,433 treatments have been administered to enrolled subjects, corresponding to a total of 26,866 syringes having been prepared. Importantly, subjects have received > 90 % of planned treatments; demonstrating strict adherence to the rigorous research protocol. For each infant, once enrolled, we carefully calculate the number of “planned doses” to be given. This calculation is based on the infant’s gestational age at birth and the date the infant will reach 32 weeks CGA. On a daily basis, we track the number of treatments that are given. Each dose that is administered is documented in the infant’s intake flow-sheet, in the medication administration record, and also in a study-specific form. Bedside nurses are blind to group assignment and document the “dose” administered as “study substance.” At the completion of the extended treatment period, the percent of “planned doses” that were actually given, is calculated and recorded. Completed treatment is defined as (the infant) having received 70 % of planned doses.

All doses have been well-tolerated by enrolled infants and no adverse effects have been reported. Infant safety is carefully assessed as described in the previous section.

We are recruiting infants from five large tertiary neonatal centers. We anticipate a racially, ethnically and economically diverse cohort, with broad representation of medically underserved women and minorities, and sufficient numbers from which to recruit and test our aims.

If aims are achieved, this study will set the standard for a consistent technique so that risks are minimized and patient safety is ensured. The evidence derived from this current study, whether positive or negative, will quickly impact neonatal clinical practice nationwide.

### Dissemination policy

Sharing of data generated by this project will be an essential part of this trial and will be carried out in several different ways. We would wish to make our results available to both neonatal clinicians (physicians, nurses, dieticians, and lactation consultants, among others) and the community of scientists interesting in improving outcomes for extremely premature infants. Our plan includes the presentation of results at national scientific meetings. We will also submit several manuscripts for publication in peer-reviewed medical and nursing journals, based on the data generated from this project.

## Trial status

This is a current, ongoing trial which is actively recruiting subjects. We expect to finish patient recruitment in September 2018 and will present final results over the course of 2019.

## Abbreviations

AIC: Akaike Information Criterion
CGA: corrected gestational age
CMV: cytomegalovirus
DSMB: Data and Safety Monitoring Board
EFW: estimated fetal weight
ELBW: extremely low birth weight
ELISA: enzyme-linked immunosorbent assay
EP: extremely premature
FDA: Food and Drug Administration
FR: free radicals
HIPAA: Health Insurance Portability and Accountability
HR: heart rate
IRB: Institutional Review Board
ITT: intention-to-treat
L-OS: late-onset sepsis
MAR: missing at random
MCAR: missing completely at random
MRM: multiple reaction monitoring
NEC: necrotizing enterocolitis
NGT: nasogastric tube
NICU: neonatal intensive care unit
OFALT: oropharyngeal-associated lymphoid tissue
OMM: own mother’s milk
OR: odds ratio
PI: principal investigator
RCT: randomized controlled trial
RR: respiratory
SQL: Structural Query Language
